# 2893 B. Effect of Cefepime versus Piperacillin-Tazobactam in Adults Hospitalized with Acute Infection: A Randomized Clinical Trial

**DOI:** 10.1093/ofid/ofad500.2477

**Published:** 2023-11-27

**Authors:** Edward Qian, Jonathan Casey, Adam Wright, Li Wang, Matthew Shotwell, Justin Siemann, Mary Lynn Dear, Joanna Stollings, Bradley Lloyd, Tanya Marvi, Kevin Seitz, George E Nelson, Patty W Wright, Edward Siew, Bradley Dennis, Jesse Wrenn, Jonathan Andereck, Jin Han, Wesley H Self, Matthew Semler, Todd Rice

**Affiliations:** Vanderbilt University Medical Center, Nashville, TN; Vanderbilt University Medical Center, Nashville, TN; Vanderbilt University Medical Center, Nashville, TN; Vanderbilt University Medical Center, Nashville, TN; Vanderbilt University Medical Center, Nashville, TN; Vanderbilt University Medical Center, Nashville, TN; Vanderbilt University Medical Center, Nashville, TN; Vanderbilt University Medical Center, Nashville, TN; Vanderbilt University Medical Center, Nashville, TN; Vanderbilt University Medical Center, Nashville, TN; Vanderbilt University Medical Center, Nashville, TN; Vanderbilt University Medical Center, Nashville, TN; Vanderbilt University Medical Center, Nashville, TN; Vanderbilt University Medical Center, Nashville, TN; Vanderbilt University Medical Center, Nashville, TN; Vanderbilt University Medical Center, Nashville, TN; Vanderbilt University Medical Center, Nashville, TN; Vanderbilt University Medical Center, Nashville, TN; Vanderbilt University Medical Center, Nashville, TN; Vanderbilt University Medical Center, Nashville, TN; Vanderbilt University Medical Center, Nashville, TN

## Abstract

**Background:**

Cefepime and piperacillin-tazobactam are commonly administered to hospitalized adults for empiric treatment of infection. Although piperacillin-tazobactam has been hypothesized to cause acute kidney injury and cefepime has been hypothesized to cause neurological dysfunction, their comparative safety has never been evaluated in a randomized trial. We aimed to determine whether choice between piperacillin-tazobactam and cefepime affects risks of acute kidney injury or neurological dysfunction.

**Methods:**

The Antibiotic Choice On ReNal outcomes (ACORN) study was a pragmatic randomized trial comparing piperacillin-tazobactam versus cefepime, conducted from November 2021 to October 2022; patient follow up concluded on November 4, 2022. Eligible patients were adults in the emergency department or medical intensive care unit for whom a clinician initiated anti-pseudomonal antibiotics within 12 hours of presentation to the hospital. Patients were randomized in a 1:1 ratio to cefepime or piperacillin-tazobactam. The primary outcome was the highest stage of acute kidney injury or death by day 14, measured on a 5-level ordinal scale ranging from no acute kidney injury to death. The two secondary outcomes were the incidence of major adverse kidney events at day 14 and number of days alive and free of delirium and coma within 14 days.

**Results:**

Among the 2,511 patients in the primary analysis (median age, 58 years; 42.7% female; 94.7% enrolled in the emergency department, 77.2% receiving vancomycin at enrollment), the highest stage of acute kidney injury or death did not differ between the cefepime and piperacillin-tazobactam groups (odds ratio [OR], 0.95; 95% confidence interval [CI], 0.80 to 1.13; P = 0.56). The incidence of major adverse kidney events at day 14 did not differ between groups (OR, 1.18; 95%CI, 0.90 to 1.54). Patients in the cefepime group experienced fewer days alive and free of delirium and coma within 14 days (OR, 0.79; 95%CI, 0.65 to 0.95).

**Figure 1.**
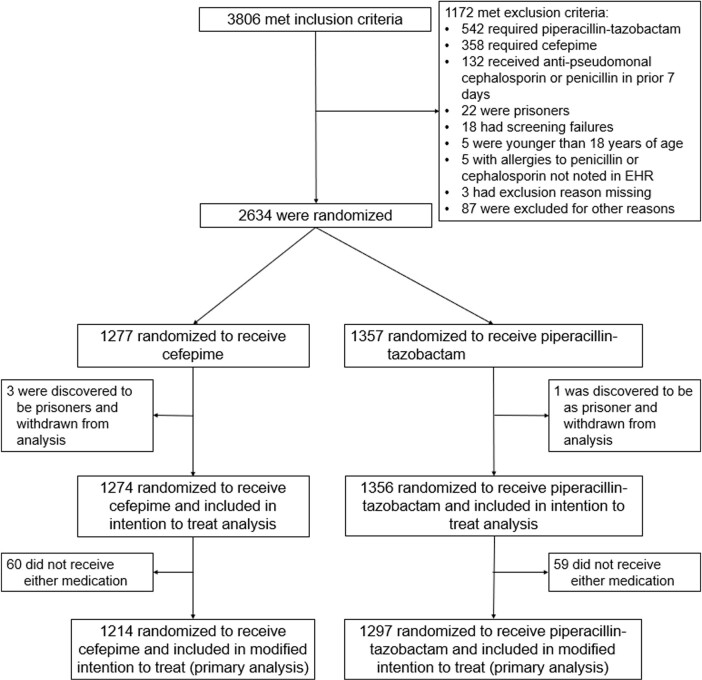
Flow of participants through the trial.

**Figure 2.**
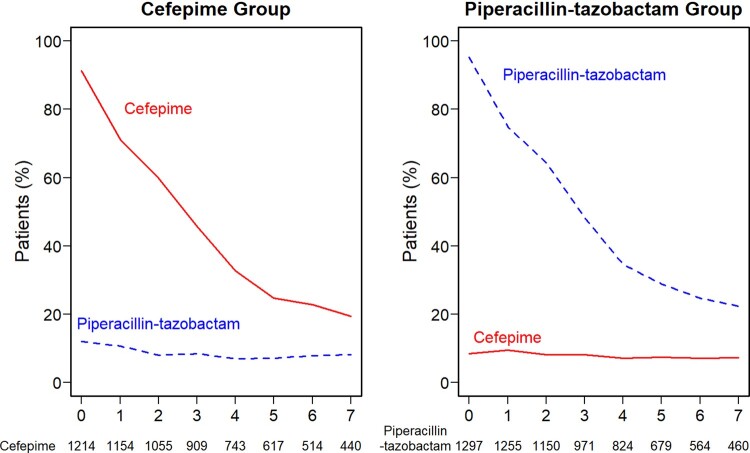
Antibiotic receipt by group

For the cefepime group (left) and piperacillin-tazobactam group (right), the percentage of patients who received cefepime (solid red) and piperacillin-tazobactam (dashed blue) is displayed on each day from enrollment through day 7. The denominator for each study day is the total number of patients who remain in the hospital.

**Figure 3:**
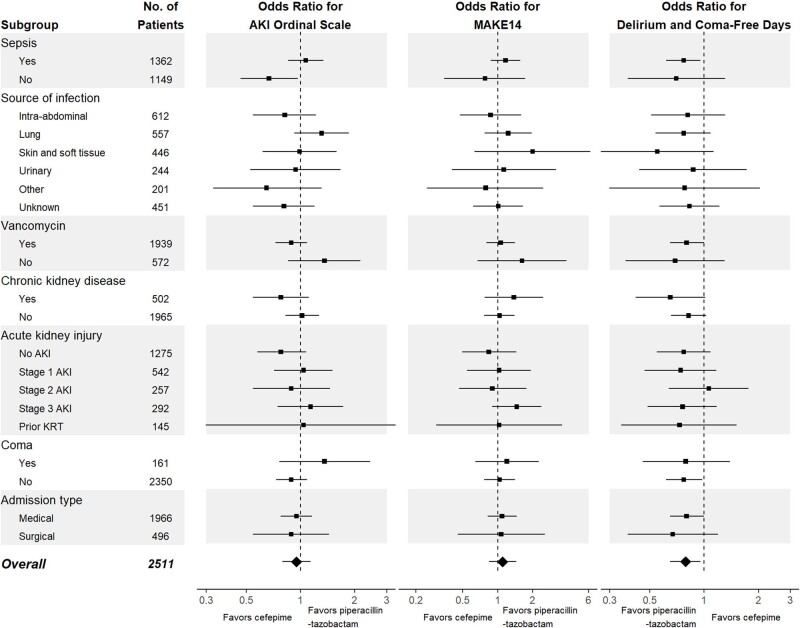
Effect Modification of the Primary and Secondary Outcomes.

For each subgroup, the effect of cefepime vs piperacillin-tazobactam is shown for the primary outcome of highest stage of acute kidney injury (AKI) or death and the secondary outcomes of major adverse kidney events at 14 days (MAKE14) and the number of days alive and free of delirium and coma within14 days. For the renal outcomes, odds ratios less than 1.0 indicate a better outcome in the cefepime group compared to the piperacillin-tazobactam group. For the outcome days alive and free of delirium and coma, an odds ratio greater than 1.0 indicates a better outcome in the cefepime group compared to the piperacillin-tazobactam group. Baseline Coma was added post hoc. KRT is kidney replacement therapy.

**Conclusion:**

Among hospitalized adults in this randomized trial, treatment with piperacillin-tazobactam did not increase the incidence of acute kidney injury or death. Treatment with cefepime resulted in more neurological dysfunction

**Disclosures:**

**Jonathan Casey, MD. MSc**, Fisher and Paykel: Travel grant **Jesse Wrenn, MD, PhD**, Bristol Myers Squibb: Grant/Research Support **Matthew Semler, MD, MSc**, Baxter International: Advisor/Consultant **Todd Rice, MD, MSc**, Cumberland Pharmaceuticals: Advisor/Consultant|Cytovale Inc: Advisor/Consultant|Sanofi: Advisor/Consultant

